# Ursodeoxycholic acid counteracts celecoxib in reduction of duodenal polyps in patients with familial adenomatous polyposis: a multicentre, randomized controlled trial

**DOI:** 10.1186/1750-1172-8-118

**Published:** 2013-08-06

**Authors:** Bjorn WH van Heumen, Hennie MJ Roelofs, M Elisa Vink-Börger, Evelien Dekker, Elisabeth MH Mathus-Vliegen, Jan Dees, Jan J Koornstra, Alexandra MJ Langers, Iris D Nagtegaal, Ellen Kampman, Wilbert HM Peters, Fokko M Nagengast,

**Affiliations:** 1 Departments of Gastroenterology & Hepatology, Radboud University Nijmegen Medical Centre, Geert Grooteplein-Zuid 10, 6525 GA, Nijmegen, The Netherlands; 2 Departments of Pathology, Radboud University Nijmegen Medical Centre, Geert Grooteplein-Zuid 10, 6525 GA, Nijmegen, The Netherlands; 3 Department of Gastroenterology & Hepatology, Academic Medical Centre, Meibergdreef 9, 1105 AZ, Amsterdam, The Netherlands; 4 Department of Gastroenterology & Hepatology, Erasmus Medical Centre,‘s-Gravendijkwal 230, 3015 CE, Rotterdam, The Netherlands; 5 Department of Gastroenterology & Hepatology, University Medical Centre Groningen, Hanzeplein 1, 9713 GZ, Groningen, The Netherlands; 6 Department of Gastroenterology & Hepatology, Leiden University Medical Centre, Albinusdreef 2, 2333 ZA, Leiden, The Netherlands; 7 Departments of Health Evidence, Radboud University Nijmegen Medical Centre, Geert Grooteplein- Noord 21, 6525 EZ, Nijmegen, The Netherlands

**Keywords:** Familial adenomatous polyposis, Chemoprevention, Celecoxib, Ursodeoxycholic acid, Duodenal adenomatosis, Cell proliferation, Apoptosis, Cyclooxygenase-2

## Abstract

**Background:**

Due to prophylactic colectomy, mortality in patients with familial adenomatous polyposis (FAP) has changed, with duodenal cancer currently being the main cause of death. Although celecoxib reduces duodenal polyp density in patients with FAP, its long-term use may increase the risk of cardiovascular events and alternatives need to be explored. Preclinical studies suggest that the combination of celecoxib with ursodeoxycholic acid (UDCA) is a potentially effective strategy. We performed a randomized, double-blind, placebo-controlled trial to investigate the effect of celecoxib and UDCA co-treatment on duodenal adenomatosis in patients with FAP.

**Methods:**

Patients with FAP received celecoxib (400 mg twice daily) and UDCA (1000-2000 mg daily, ~20-30 mg/kg/day, n=19) or celecoxib and placebo (n=18) orally for 6 months. Primary outcome was drug efficacy, assessed by comparing duodenal polyp density at pre- and post-intervention by blinded review of endoscopic recordings. As secondary outcomes, cell proliferation, apoptosis, and COX-2 levels in normal duodenal mucosa were assessed by immunohistochemistry or real-time quantitative polymerase chain reaction.

**Results:**

In intention-to-treat analysis, deceased polyp density was observed after celecoxib/placebo treatment (p=0.029), whereas increased polyp density was observed after celecoxib/UDCA treatment (p=0.014). The difference in change in duodenal polyp density was statistically significant between the groups (p=0.011). No changes in secondary outcomes were observed. Thirty patients (81%) reported one or more adverse events, 16 patients (84%, Common Toxicity Criteria for Adverse Events version 3.0 (CTCAE) grade 1–3) treated with celecoxib/UDCA and 14 patients (78%, CTCAE grade 1–2) treated with celecoxib/placebo. Nine patients (24%) discontinued intervention prematurely, 5 patients (26%) treated with celecoxib/UDCA and 4 patients (22%) treated with celecoxib/placebo.

**Conclusions:**

Celecoxib reduces duodenal polyp density in patients with FAP, and unexpectedly, high dose UDCA co-treatment counteracts this effect. The benefit of long term use of celecoxib for duodenal cancer prevention needs to be weighed against the (risk of) adverse events.

**Trial registration:**

http://ClinicalTrials.gov, identifier NCT00808743

## Background

In the past decades, prophylactic colectomy to prevent development of colorectal cancer substantially improved prognosis in patients with familial adenomatous polyposis (FAP; OMIM #175100) [1]. The mortality pattern has changed and duodenal cancer now is the main cancer-related cause of death [2,3]. Lifetime risk of duodenal adenomas approaches 100% [4], and approximately 3-7% of patients develop duodenal cancer [5,6]. As duodenal cancer in patients with FAP has a poor prognosis [7,8], the clinical challenge is to identify patients with high-risk duodenal adenomas and intervene before progression to cancer occurs. Prophylactic duodenectomy may offer a prolonged disease-free interval, but is associated with substantial morbidity and mortality [9,10]. Therefore, chemoprevention would be highly desirable to postpone or even avoid the necessity for radical surgery.

Cyclooxygenase (COX) inhibiting non-steroidal anti-inflammatory drugs (NSAIDs) have been investigated extensively as potential chemopreventive drugs. COX-2 is induced in inflammatory and tumorigenic settings [11]. Overexpression of COX-2, as found in colorectal adenomas and carcinomas, was linked to reduced apoptosis, enhanced cell growth, tumour angiogenesis, tissue invasion, and metastasis [11]. Treatment with the COX-2 inhibitor celecoxib resulted in regression of colorectal adenomas in patients with FAP [12], as well as in significant decrease in sporadic colorectal adenomas [13,14].

For duodenal polyposis, the value of COX inhibiting agents is not yet established [15]. Sulindac showed regression of small duodenal polyps in patients with FAP [16,17], but had no benefit in controlling periampullary polyposis [18]. The significant reduction in duodenal polyp density after 6 months of treatment with high dose celecoxib in patients with FAP with clinically significant disease was promising [19].

Unfortunately, suitability of COX-2 inhibitors for long-term use is subject of discussion, due to increased risks of adverse cardiovascular events [13,14,20]. Combining celecoxib with other potentially effective drugs could be a more effective strategy. A candidate drug is ursodeoxycholic acid (UDCA), for a number of reasons. First, the clustering of adenomas around the ampulla of Vater suggests that bile plays a role in duodenal adenomatosis [21]. In *in vitro* models of human colorectal cancer cells, UDCA significantly reduced cytotoxicity of secondary bile acids [22], and celecoxib and UDCA co-treatment inhibited cell growth in colorectal adenoma cells from a patient with FAP [23]. Second, clinical studies showed chemopreventive effects of UDCA on development of colorectal neoplasms, in patients with sporadic colorectal adenomas, and in patients with ulcerative colitis (UC) and primary sclerosing cholangitis (PSC) [24-26]. Third, UDCA was found to suppress COX-2 levels in a rat model of colonic carcinogenesis [27], suggesting an alternative pathway for COX-2 inhibition [28]. Finally, in a mouse model of FAP, sulindac and UDCA co-treatment showed synergistic effects in the prevention of intestinal adenomas [29].

Based on these findings, the aim of the present randomized controlled trial was to examine the effect of celecoxib plus UDCA co-treatment, in comparison to celecoxib plus placebo, on duodenal adenomatosis in patients with FAP. We hypothesized that adding UDCA to the treatment with celecoxib results in a further reduction of duodenal polyp density.

## Patients and methods

This clinical trial (http://ClinicalTrials.gov number NCT00808743) was conducted according to ICH Good Clinical Practice and complied with the principles of the amended Declaration of Helsinki and Dutch legislation. Ethical approval was obtained at the initiating centre Radboud University Nijmegen Medical Centre (RUNMC; Protocol approval number 2008/148; CCMO number NL23569.091.08). In the other participating centres, feasibility was approved by the local Medical Ethics Committees. All study participants provided written informed consent. The study was monitored by a RUNMC Safety Monitoring Board.

### Study participants

The study population consisted of patients with FAP recruited from the cohort under regular surveillance at the RUNMC, Academic Medical Centre Amsterdam (AMC), Erasmus Medical Centre Rotterdam (EMC), University Medical Centre Groningen (UMCG), and Leiden University Medical Centre (LUMC). The study was conducted between June 2009 and June 2011.

The diagnosis FAP was established either clinically, by the presence of >100 colorectal polyps, or genetically, by the presence of adenomatous polyposis coli (*APC*) gene mutations. Eligible patients were between 18 and 70 years of age, capable of informed consent, had Spigelman stage II or III duodenal adenomatosis at last surveillance duodenoscopy, and had no history of surgical duodenal resection. Exclusion criteria included peptic ulcer disease, inflammatory bowel disease, cardiovascular disease (congestive cardiac failure with New York Heart Association class ≥II; history of ischemic heart disease and/or cerebrovascular disease) or significant cardiovascular risk (at least two of the following risk factors: hypertension, hypercholesterolaemia, diabetes mellitus, ≥2 first degree relatives with cardiovascular event below the age of 55 years), abnormal results on a full blood count or abnormal liver or renal function tests, known intolerability of NSAIDs, sulfonamids, or UDCA, use of NSAIDs or UDCA for >1 week during 6 months prior to study entry, use of lithium, and pregnancy or lactation.

### Study procedures

Evaluation at baseline included history taking, physical examination, and clinical laboratory evaluation (full blood count, liver and renal function, cholesterol). Endoscopic procedures were performed using a side-viewing endoscope (Olympus TJF-160, Olympus Medical Systems Europe, Hamburg, Germany) and a forward-viewing endoscope (Olympus GIF-1T-Q160) successively. Endoscopic procedures were recorded digitally. After completion of the recording procedures, six random biopsies of normal appearing mucosa were taken in the second (D2) portion of the duodenum. Two biopsies were fixed in formalin and embedded in paraffin, four biopsies were snap frozen in liquid nitrogen and stored at −80°C. Biopsies were taken using an Olympus Endojaw FB-232U with open forceps diameter 9mm, or a Boston Scientific Radial Jaw 3 with open forceps diameter 8mm (Boston Scientific, Natick, MA, USA). Procedures were repeated after 6 months. At baseline, no biopsies of adenomatous lesions were taken, as this could influence primary outcome.

After completion of pre-intervention duodenoscopy, patients were randomly assigned to one of two treatment groups in an 1:1 ratio. Randomization was performed at the Department of Clinical Pharmacy RUNMC, by a computer-generated schedule, to assign sequentially numbered treatment packs in randomized blocks of four. Patients, physicians, and investigators were blinded to treatment allocation. Patients in group A received orally for 6 months: celecoxib (Celebrex, Pfizer, New York, NY, USA) 400 mg twice daily (once daily during the first 2 weeks), in combination with UDCA (Ursofalk, Dr Falk Pharma, Freiburg, Germany). Patients in study group B received orally for 6 months: celecoxib 400mg twice daily (once daily during the first 2 weeks), in combination with an UDCA identical-appearing placebo (Dr Falk Pharma). UDCA/placebo was given in two daily doses, with total daily UDCA dose based on body weight: ≤50 kg: 1000 mg, 50-70 kg: 1500 mg, >70 kg: 2000 mg (~20-30 mg/kg/day). UDCA starting dose was 500 mg, which was raised with 500 mg every 2 weeks until maximum dose was reached. The placebo contained lactose and cellulose.

Information on adverse events (AEs) was obtained during patient contacts by telephone at 1 and 3 months, and prior to post-intervention duodenoscopy at 6 months. Monitoring of blood pressure and clinical laboratory parameters was performed at 1 and 6 months. AEs were graded as defined by the Common Toxicity Criteria for Adverse Events version 3.0 (CTCAE v3.0) [30]. Compliance was monitored by means of pill counts and review of diaries completed by the patients.

Disclosure of randomization was performed by the Department of Clinical Pharmacy RUNMC on December 10^th^ 2012, after completion of assessment of recorded duodenoscopies and all tissue analyses.

### Assessment of recorded endoscopic procedures

Endoscopic recordings were analyzed using qualitative assessment of duodenal polyp density, as previously described in patients with FAP for the colorectum [12] and duodenum [19]. In short, five gastroenterologists experienced in management of FAP (ED, JD, JJK, AMJL, FMN), independently scored the blinded pairs of pre- and post-intervention videos of each patient, shown in random order. Pairs were scored as no change (scored as 0), clinical improvement (scored as +1), or clinical deterioration (scored as −1) in polyp density. Based on the scores of the five gastroenterologists, mean scores of change in duodenal polyp density were calculated for each patient. Patients that discontinued intervention prematurely were included in intention-to-treat analysis with a score of change in duodenal polyp density of −0.5.

### Immunohistochemical staining for cell proliferation, apoptosis, and COX-2

Tissue sections of 4 μm were cut from paraffin blocks, mounted on electrostatic slides (Super Frost Plus, Menzel-Gläser, Baunschweig, Germany) and stained with Hematoxylin & Eosin (H&E). Only samples with normal histology (non-dysplastic and non-adenomatous mucosa), as verified by an expert pathologist (IDN), were used for further analyses.

Tissue sections were deparaffinized and dehydrated. Endogenous peroxidase was blocked with 3% hydrogen peroxide. Subsequently, heat-induced antigen retrieval was performed in sodium citrate buffer (10 mmol/L, pH=6). Cell proliferation activity was assessed after staining for 1 hour at room temperature with mouse anti-human MIB-1 monoclonal antibody (Dako A/S, Glostrup, Denmark) at dilution 1:200. MIB-1 recognizes the Ki-67 nuclear antigen of dividing cells [31]. Apoptosis was assessed by staining overnight at 4°C with mouse anti-human M30 CytoDEATH monoclonal antibody (Roche Diagnostics, Mannheim, Germany) at dilution 1:400. M30 recognizes cleaved cytokeratin 18, expressed in epithelial cells during early apoptosis [32]. COX-2 was assessed by staining overnight at 4°C with mouse anti-human COX-2 monoclonal antibody (Cayman Chemical, Ann Arbor, MI, USA) at dilution 1:100. Visualization of MIB-1 was achieved using the Brightvision (1:1)/BrightDab detection system (Immunologic, Duiven, The Netherlands), whereas M30 and COX-2 were visualized using the avidin-biotin peroxidase complex method (Vector Laboratories, Burlingame, CA, USA). Mayer hematoxylin counterstaining was applied. Tissue sections of colorectal carcinomas were used as positive controls.

### Evaluation of immunohistochemical staining and scoring

Tissue samples were independently evaluated by light microscopy (Leica Microsystems, Rijswijk, The Netherlands) by two investigators (BWHvH, MEV-B). If scores differed, a consensus agreement was reached during re-evaluation. A random selection of 10% of scores were re-evaluated and verified by an expert pathologist (IDN). Cell proliferation index was expressed as percentage of MIB-1 positive epithelial cells in areas of the tissue section with well-orientated crypt-villi architecture. Apoptotic index was expressed as number of M30 positive epithelial cells per mm^2^ tissue area. COX-2 staining in epithelial cells was scored as previously described [33]: 0, no staining; 1, weak cytoplasmatic and membranous staining (may contain strong staining in <10% of cells); 2, moderate-to-strong staining in 10-90% of cells; and 3, strong staining in >90% of cells.

### RNA isolation and real-time quantitative polymerase chain reaction (qPCR) for COX-2

One biopsy sample of each location was weighed and taken up in 200 μl TRIzol (Life Technologies, Pailey, UK). Tissue was homogenized by 10 strokes with a Teflon pestle. After homogenization, another 600 μl TRIzol was added. Total RNA was extracted according to the manufacturer’s instructions (Life Technologies) with a slight modification: prior to precipitating the RNA with isopropyl alcohol, 7.5 μg RNAse-free glycogen was added to the aqueous phase. Approximately 1 μg RNA was converted into cDNA according to the instructions provided by the Roche Transcriptor High Fidelity cDNA synthesis kit (Roche Diagnostics). Detection and quantification of COX-2 mRNA was performed by qPCR using the CFX96 Real-Time PCR Detection System (Bio-Rad Laboratories, Hercules, CA, USA). Analysis of COX-2 expression was performed using two different COX-2 specific primer sets: forward 5’-GGCGCTCAGCCATACAG-3’(exon 1) with reverse 5’-CCGGGTACAACTGCACTTAT-3’(exon 2) and forward 5’-GGCGCTCAGCCATACAG-3’(exon 1) with reverse 5’-TCTTGTCAAAAATTCCGGTG-3’ (exons 2 and 3) (Isogen Life Science, Maarssen, The Netherlands). PCR products were detected with SYBR Green (Molecular Probes, Eugene, OR, USA). Specificity of COX-2 PCR products was checked using melting curve analysis and agarose gel electrophoresis. Levels of β-2 microglobulin (β2M) mRNA were used as a normalizing control. Analysis of β2M was performed with the primers 5’-ATGAGTATGCCTGCCGTGTG- 3’ and 5’-CCAAATGCGGCATCTTCAAAC-3’ with a specific probe 5’-FAMCGCGTCGTGGGATGGAGACATGTAAGCAGACGCGDabcyl- 3’ (Biolegio, Nijmegen, The Netherlands). The β2M product was checked by agarose gel electrophoresis. qPCR procedures were performed in triplicate or quadruplicate and mean Ct values were calculated.

### Statistical analysis

Baseline characteristics were expressed as percentage or medians with range when appropriate. Continuous variables were considered to be not normally distributed. Differences between treatment groups on continuous variables were tested using Mann–Whitney U test, and differences on discrete variables were examined using Chi-square test, or Fisher’s exact test when appropriate. Differences on continuous and ordinal variables within treatment groups, comparing pre- and post-intervention measurements, were examined using Wilcoxon Signed Rank test and McNemar’s test, respectively. Analyses of primary outcome were performed on an intention-to-treat basis, with a per-protocol analysis as sensitivity analysis. P value of <0.05 (2-sided) was considered statistically significant. Statistical analysis was performed using SPSS statistical software version 21 (IBM SPSS, Chicago, IL, USA).

## Results

### Patient characteristics

**Figure 1:**
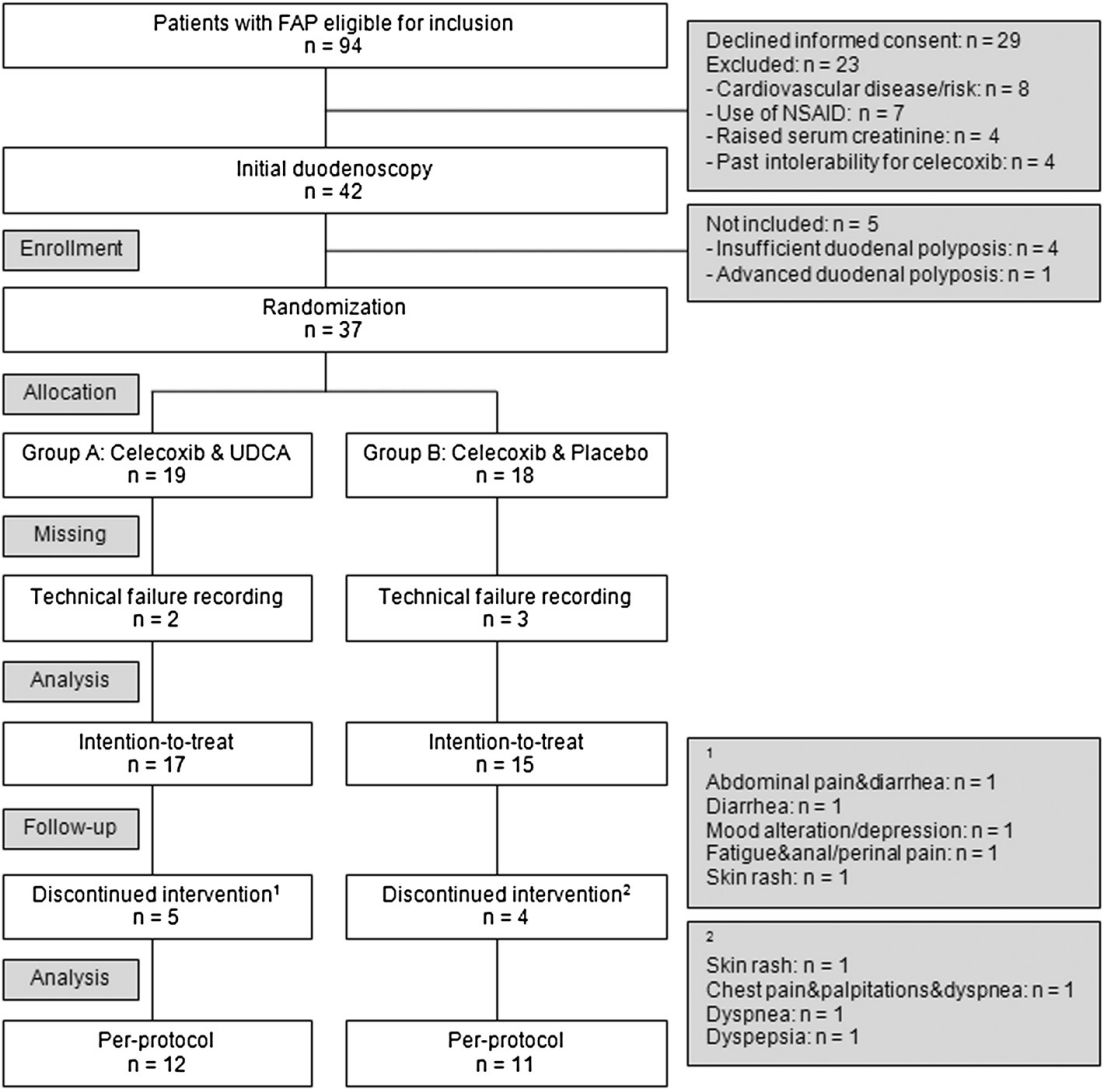
**CONSORT diagram.** FAP = familial adenomatous polyposis; NSAID = non-steroidal anti-inflammatory drugs; UDCA = ursodeoxycholic acid.

The CONSORT diagram of the study is depicted in Figure 1. Of all patients with FAP that were under regular surveillance in the five participating University hospitals, 94 patients were eligible for inclusion. Twenty-three patients were excluded based on exclusion criteria and 29 patients declined informed consent. Forty-two patients underwent initial duodenoscopy, of which five patients were not randomized: four had insufficient polyps and one patient required treatment because of advanced duodenal adenomatosis. Thirty-seven patients were randomized: 19 patients received celecoxib & UDCA (group A) and 18 patients received celecoxib & placebo (group B). Patient characteristics are depicted in Table 1. Due to technical failure, either pre- or post-intervention recordings could not be analysed in five patients. Consequently, thirty-two patients (group A, n=17; group B, n=15) were analysed on an intention-to-treat basis for the primary outcome. Nine patients (24.3%) discontinued intervention prior to duodenoscopy at six months. Consequently, per-protocol analysis was performed on 23 patients (group A, n=12; group B, n=11).

**Table 1:** Base-line characteristics of patients with FAP

	**Overall population**	**Group A:** **Celecoxib**&**UDCA**	**Group B:** **Celecoxib**&**Placebo**	**p value**
Number of patients	37	19	18	
Age at study entry, median/range (yr)	42/22–67	42/22–67	41/27–64	0.964^1^
Sex (n, %)				0.618^2^
Male	18 (48.6)	10 (52.6)	8 (44.4)	
Female	19 (51.4)	9 (47.4)	10 (55.6)	
Participants per centre (n, %)				0.932^3^
RUNMC	18 (48.6)	10 (52.6)	8 (44.4)	
AMC	10 (27.0)	4 (21.1)	6 (33.3)	
EMC	4 (10.8)	2 (10.5)	2 (11.1)	
UMCG	3 (8.1)	2 (10.5)	1 (5.6)	
LUMC	2 (5.4)	1 (5.3)	1 (5.6)	
Body Mass Index, median/range (kg/m^2^)	25.6/18.8–34.5	26.0/19.2–34.5	25.6/18.8–33.1	0.408^1^
Diagnosis FAP				0.660^3^
Clinical only	6 (16.2)	4 (21.2)	2 (11.1)	
*APC* gene mutation	31 (83.8)	15 (78.9)	16 (88.9)	
Age at primary CR surgery, median/range (yr)	21/7–60	22/7–60	18.5/11–48	0.298^1^
Time since primary CR surgery, median/range (yr)	18/1–38	17/1–33	20.5/8–38	0.178^1^
Type of primary CR surgery				0.738^3^
IRA	18 (48.6)*	10 (52.6)*	8 (44.4)	
IPAA	14 (37.8)	6 (31.6)	8 (44.4)	
Ileostomy	5 (13.5)	3 (15.8)	2 (11.1)	
Secondary CR surgery (n, %)	11 (29.7)	5 (26.3)	6 (33.3)	0.641^2^
Spigelman stage at last surveillance before entry				0.985^2^
II	19 (51.4)**	10 (52.6)	9 (50)**	
III	17 (45.9)**	9 (47.4)	8 (44.4)**	

* Including one patient who underwent ileosigmoid anastomosis; ** In 1 case exact data on last previous surveillance duodenoscopy was missing; ^1^ The P value was calculated using the Mann–Whitney U test; ^2^ The P value was calculated using the chi-square test; ^3^ The P value was calculated using the Fisher’s exact test. *FAP* familial adenomatous polyposis, *APC* adenomatous polyposis coli, *CR* colorectal, *IRA* ileorectal anastomosism, *IPAA* ileal pouch-anal anastomosis; Ileostomy: proctocolectomy with ileostomy, *UDCA* ursodeoxycholic acid, *AMC* Academic Medical Centre Amsterdam, *EMC* Erasmus Medical Centre Rotterdam, *LUMC* Leiden University Medical Centre, *RUNMC* Radboud University Nijmegen Medical Centre, *UMCG* University Medical Centre Groningen.

### Primary outcome: change in duodenal polyp density

**Figure 2:**
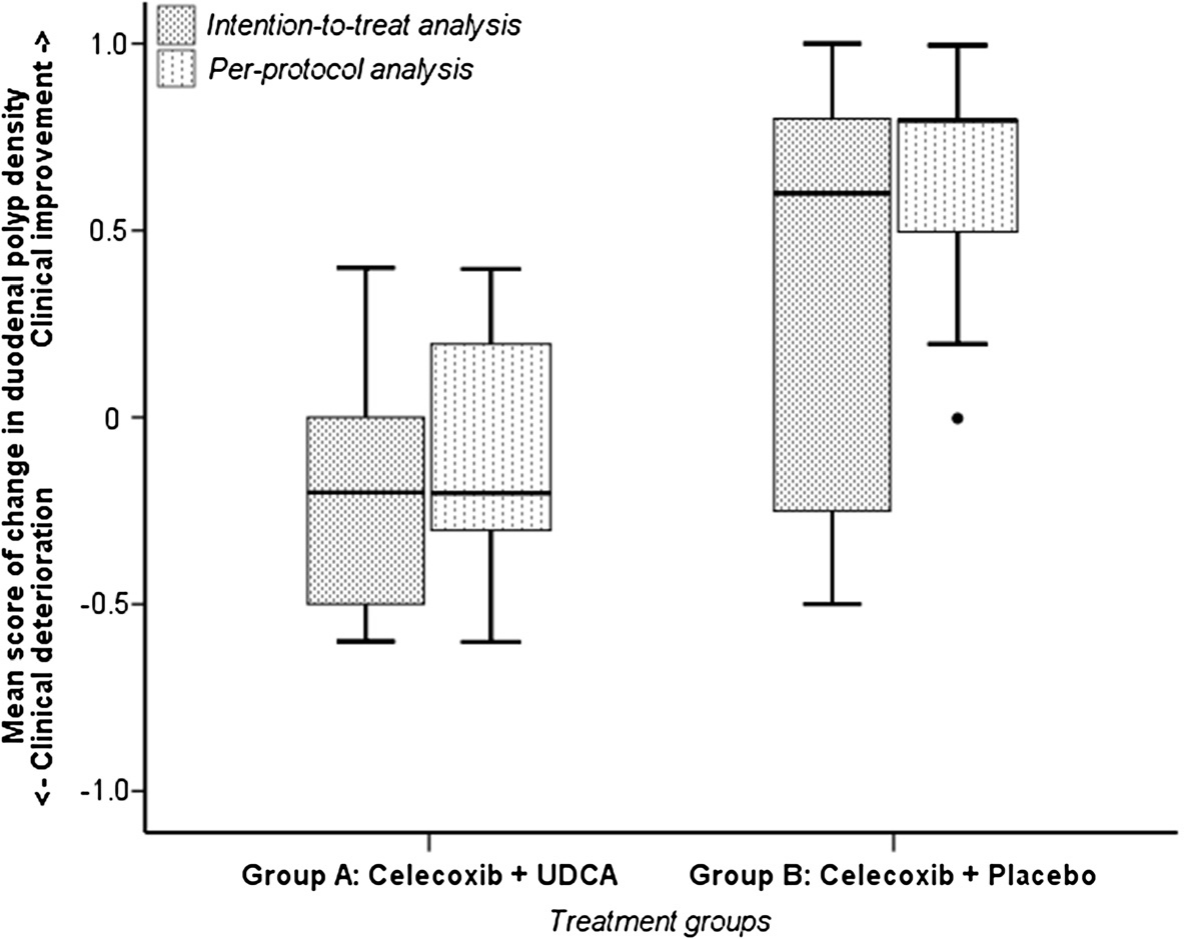
**Box-Whisker plots of intention-to-treat and per-protocol analysis.** Intention-to-treat analysis of mean score of change in duodenal polyp density comparing duodenoscopic recordings pre- and post-intervention with either celecoxib & UDCA (group A) or celecoxib & placebo (group B): clinical deterioration in group A (n=17, Wilcoxon Signed Rank, p=0.014), clinical improvement in group B (n=15, Wilcoxon Signed Rank, p=0.029); difference in mean score between groups statistically significant (Mann–Whitney U, p=0.011). Per-protocol analysis: clinical deterioration in group A (n=12, Wilcoxon Signed Rank, p=0.271), clinical improvement in group B (n=11, Wilcoxon Signed Rank, p=0.004); difference in mean score between groups statistically significant (Mann–Whitney U, p<0.001). UDCA = ursodeoxycholic acid.

In the intention-to-treat analysis, clinical deterioration (n=17, median= −0.2, range: -0.6-+0.4) in duodenal polyp density was observed in group A (Wilcoxon Signed Rank, p=0.014), receiving celecoxib & UDCA, while clinical improvement (n=15, median=0.6, range: -0.5-+1.0) was observed in group B (Wilcoxon Signed Rank, p=0.029), receiving celecoxib & placebo (Figure 2). The difference in mean score of change in duodenal polyp density was statistically significant between groups (Mann–Whitney U, p=0.011).

In the per-protocol analyses, the difference in mean score of change in duodenal polyp density between group A (n=12, median=−0.2, range: -0.6-+0.4) and group B (n=11, median=0.8, range: 0.0-+1.0) was more pronounced (Mann–Whitney U, p<0.001). Clinical deterioration in duodenal polyp density observed in group A was not statistically significant (Wilcoxon Signed Rank, p=0.271), in contrast to the clinical improvement observed in group B (p=0.004).

### Secundary outcome: cell proliferation, apoptosis, and COX-2 immunohistochemistry

Changes in cell proliferation, apoptosis, and COX-2 were evaluated in all patients that completed the intervention period and underwent pre- and post-intervention duodenoscopy, with one additional patient excluded in group B of whom post-intervention biopsies could not be assessed (n=27).

Median difference in cell proliferation pre- versus post-intervention was not statistically significant between both treatment groups (group A: n=14, median difference =−5.0%, range=−20.0%-10.0%; group B: n=13, median difference=0.0%, range: -15.0%-20.0%; Mann–Whitney U, p=0.141). The median decrease in cell proliferation of 5.0% observed in group A was not statistically significant (Wilcoxon Signed Rank, p=0.057).

No M30 positive apoptotic epithelial cells were scored in any of the evaluated samples, except for positive control samples.

COX-2 staining was scored as either moderate-to-strong or strong staining in all evaluated samples. No difference in COX-2 staining was seen comparing pre- and post-intervention: in group A, a decreased score was observed in 2 patients, an equal score in 8 patients, and an increased score in 3 patients (McNemar, p=1.000). In group B, a decreased score was observed in 4 patients, an equal score in 6 patients, and an increased score in 2 patients (McNemar, p=0.688).

### Secondary outcome: COX-2 mRNA analyses

COX-2 mRNA expression was also evaluated in all patients that completed the intervention period, with the noted one additional patient excluded in group B (n=27).

In 12 patients (group A: n=6, group B: n=6), no measurable COX-2 mRNA levels were present in either pre- or post-intervention sample. In all other cases, low COX-2 mRNA levels seemed present, but specificity of PCR products could not be confirmed by melting curve analyses and agarose gel electrophoresis. Experiments in which the second set of specific COX-2 primers were used, showed the same results. Simultaneous qPCR analyses on colorectal cancer tissue samples showed high levels of COX-2 specific qPCR products.

### Adverse events and compliance

AEs were analysed for all randomized patients (n=37). An overview of all 58 AEs reported by 30 patients (81.1%) is shown in Table 2. In group A (n=19), 10 grade 1, 18 grade 2, and 6 grade 3 AEs were reported by 16 patients (84.2%), whereas in group B (n=18), 9 grade 1, and 15 grade 2 AEs were reported by 14 patients (77.8%) (Fisher’s exact, p=0.114). Five patients (26.3%) discontinued intervention in group A, due to complaints of abdominal pain and diarrhea (n=1), diarrhea (n=1), mood alteration/depression (n=1), fatigue and anal/perianal pain (n=1), and skin rash (n=1). Four patients (22.2%) discontinued intervention in group B, due to complaints of skin rash (n=1), chest pain, palpitations, and dyspnea (n=1), dyspnea (n=1), and dyspepsia (n=1) (Fisher’s exact, p=1.000). Two patients in group A reported insomnia and edema of the lower limbs respectively, which resolved after reducing the celecoxib dose to halve the standard trial dose. Both patients completed the intervention period and were included in the analyses.

**Table 2:** **Adverse events in patients with FAP treated with either celecoxib** &**ursodeoxycholic acid or celecoxib** &**placebo**

		**Treatment groups**	
**CTCAE Category**	**Name adverse event**	**Group A** **n = 19**	**Group B** **n = 18**
Auditory/Ear	Otitis, middle ear	1 (0/1/0)	0
Blood/Bone marrow	Anemia - hemoglobin	1 (1/0/0)	0
	Leukopenia	1 (0/1/0)	0
Cardiac arrhythmia	Palpitations	0	2 (2/0/0)
Constitutional symptoms	Fatique	2 (0/1/1)	1 (0/1/0)
	Insomnia	1 (0/0/1)	0
Dermatology/Skin	Hair loss - scalp	1 (1/0/0)	0
	Rash	1 (0/1/0)	1 (0/1/0)
Gastrointestinal	Constipation	2 (2/0/0)	2 (2/0/0)
	Diarrhea	2 (1/1/0)	2 (2/0/0)
	Heartburn/dyspepsia/nausea	4 (1/3/0)	2 (1/1/0)
	Ulcera - oral	0	1 (1/0/0)
	Ulcera - ileum/colon/rectum	1 (1/0/0)	0
Hepatobiliary/Pancreas	Pancreas irritation*	0	1 (0/1/0)
Infection	Infection - gastroenteritis	1 (0/1/0)	2 (0/2/0)
	Infection - dental-tooth	1 (0/1/0)	1 (0/1/0)
	Infection - skin	1 (0/1/0)	1 (0/1/0)
Lymphatics	Edema - lower limbs	2 (1/1/0)	0
Metabolic/Laboratory	Elevated AST, GGT	1 (1/0/0)	1 (1/0/0)
	Hypokalemia	1 (0/0/1)	0
Neurology	Dizzyness	1 (1/0/0)	0
	Mood alteration - depression	1 (0/0/1)	0
	Neuropathy - carpal tunnel syndrome	1 (0/1/0)	0
Pain	Abdominal	1 (0/0/1)	0
	Anal/perianal	4 (0/4/0)	1 (0/1/0)
	Joint	0	1 (0/1/0)
	Chest/thorax	0	1 (0/1/0)
Pulmonary/Upper respiratory	Dyspnea	0	2 (0/2/0)
	Nasal cavity/paranasal sinus reaction	0	2 (0/2/0)
Renal/Genitourinary	Lower urinary tract symptoms - prostatism	1 (0/1/0)	0
Secondary malignancy	Secondary malignancy - basalioma - nose	1 (0/0/1)	0
	Total n of reported AE	34 (10/18/6)**	24 (9/15/0)**
	Patients reporting ≥1 AE (n, %)	16 (84.2%)	14 (77.8%)

Number of specific adverse event reported during 6 month intervention in patients with FAP with either celecoxib & ursodeoxycholic acid (Group A) or celecoxib & placebo (Group B), is depicted as grade 1, 2, or 3, as defined by the Common Terminology Criteria for Adverse Events (CTCAE) version 3.0. Grade 4 and 5 adverse events did not occur. * Adverse event related to pre-intervention duodenoscopy; no other adverse events related to duodenoscopy were reported; ** Distribution of number of adverse events grade 1, 2, or 3, was not significantly different between treatment groups (Fisher’s exact, p=0.114). *CTCAE* Common Terminology Criteria for Adverse Events, *FAP* familial adenomatous polyposis, *AST* aspartate aminotransferase, *GGT* gamma-glutamyl transpeptidase, *AE* Adverse Event.

Compliance was evaluated in all patients that completed the 6 months intervention period (n=28). In group A, the median compliance for celecoxib and UDCA was 98.4% (range: 82.4-100%) and 96.8% (range: 42.2-100%), respectively. In group B, the median compliance for celecoxib and placebo was 99.4% (range: 79.1-100%) and 97.0% (range: 80.3-100%), respectively.

## Discussion

This randomized controlled trial confirms that celecoxib mono-treatment reduces duodenal polyp density in patients with FAP, whereas it demonstrates that celecoxib and UDCA co-treatment has no beneficial effect. In contrast to our hypothesis of an expected additional effect of the combination, our results imply that the clinical improvement observed in patients treated with celecoxib alone is counteracted by co-treatment with UDCA. We found no changes in cell proliferation, apoptosis or COX-2 expression in normal duodenal mucosa of patients with FAP, that could explain the observed effects.

The clinical improvement of duodenal polyp density after treatment with celecoxib alone, confirms results from a previous trial with similar design [19]. COX-2 overexpression was found in oesophageal [34], gastric [35], colorectal [36], as well as small intestinal cancer [37]. Multiple lines of evidence, including results from *in vitro*, animal, and clinical studies, indicated that inhibition of the increased COX-2 expression, at least in part accounts for the anti-proliferative activity of celecoxib [38]. In addition, COX-2 independent pathways were suggested to be involved in the anti-proliferative effect of celecoxib [38,39]. To our surprise, we found a high COX-2 expression by immunohistochemical analyses, but detected no COX-2 mRNA expression in normal appearing duodenal mucosa of patients with FAP, neither pre- nor post-intervention. Consequently, controversy exists between assessment of COX-2 by immunohistochemistry or qPCR assay. We assume that results on COX-2 in immunohistochemistry could be based on aspecific protein staining by the COX-2 antibody. Assessment of duodenal COX-2 mRNA levels by using Quantigene Plex Assay, confirmed our findings with the qPCR assay: COX-2 mRNA expression is extremely low or even absent in normal duodenal mucosa of patients with FAP (van Heumen *et al.*, manuscript in preparation). Our results are in agreement with a previous report of undetectable COX-2 mRNA levels in human small intestinal mucosa by using qPCR analysis [37].

After prophylactic colectomy, duodenal bile composition changes and largely consists of cholic acid (CA) and chenodeoxycholic acid (CDCA) [40]. In *in vitro* models of human colon cancer cells, UDCA significantly reduced cytotoxicity of secondary bile acids [22]. By UDCA supplementation in patients with FAP, up to 50% enrichment of duodenal bile with UDCA was reached, with a large reduction in concentration of the cytotoxic CDCA [41]. Based on these findings, an inhibition of cell proliferation was expected after UDCA supplementation. Although we combined celecoxib and high dose UDCA (~20-30 mg/kg daily), these *in vitro* effects could not be reproduced *in vivo* in our trial. Moreover, our hypothesis was in part based on clinical studies in patients with UC and PSC showing chemopreventive effects of UDCA on development of colorectal neoplasms [25,26]. Recently however, treatment of patients with UC and PSC with high dose UDCA (28-30 mg/kg daily) was found to be associated with an increased risk of colorectal neoplasms [42]. This could be an explanation for the disappointing effect we obtained by the combination treatment of celecoxib and high dose UDCA. In contrast, a recent meta-analysis revealed that long-term low dose UDCA treatment (8-15mg/kg daily) reduces the risk of advanced colorectal neoplasms in patients with UC and PSC [43]. Extrapolating these results, long-term low dose UDCA treatment could be expected to be effective in patients with advanced duodenal adenomatosis. However, in a clinical trial in patients with FAP, no effects of low dose UDCA (10 mg/kg daily) after 24 months as mono-treatment were found on Spigelman scores [44]. Future studies that focus on the intracellular mechanisms of action may elucidate the ambivalent effect of UDCA as chemopreventive drug in (pre-) clinical studies.

The present study has several strenghts. First, it is the first randomized clinical trial to investigate a combination of two potential chemopreventive drugs for duodenal adenomatosis in patients with FAP. Second, the study population consists of an unique sample of patients with FAP from 5 out of the 8 Dutch University Medical Centres. Third, bias due to interobserver variability was minimized, as primary outcome was based on scores of polyp density by 5 gastroenterologists, who independently compared pre- and post-intervention videos shown in random order, while blinded to treatment allocation. The following limitations are noted. First, our study lacks a ‘true placebo’ group. Hence, we were not able to confirm the spontaneous reduction in duodenal polyps in the placebo group, that was previously described [19,44]. Second, changes in duodenal polyp density are assessed qualitatively. In previous chemopreventive studies on colorectal adenomatosis, changes in polyp density were assessed by exact counting of polyp number and measuring polyp diameter [12,45]. This method is not suitable for assessment of the plaque-like duodenal polyps, which are partially obscured due to folding over the mucosal folds. Moreover, the curved anatomy of the duodenum introduces an optic bias in the two dimensional images obtained during endoscopy, which further hampers reliable quantification. In clinical practice, the Spigelman scoring system is an established tool to assess duodenal adenomatosis and is commonly used to plan follow up or treatment [4,46]. In clinical science however, the Spigelman score seems insufficiently distinctive to detect subtle changes in polyp density, and it does not account for peri-ampullary adenomatosis specifically [47]. The applied method of assessment in our study, which does include visual assessment of the peri-ampullary region by side-viewing duodenoscopy, permits adequate comparison with previous studies in the field [12,19,45]. Third, although we were able to detect a significant difference in change in duodenal polyp density between the two treatment groups, sample size is fairly small. As the participants already were under regular endoscopic surveillance and a chemopreventive option to their benefit was the aim of our study, we expected a high willingness to participate in the trial. However, of all eligible patients with FAP under regular surveillance in any of the five participating centres, 31% declined informed consent. Reports of cardiotoxicity of celecoxib [13,14,20] could have withheld patients with FAP to participate. In addition, because of these reports, we applied strict exclusion criteria, leaving out another 24% of patients. We seemingly underestimated the required dedication to participate in the strenuous study protocol, which included a relatively short follow-up interval of 6 months, as compared to regular surveillance intervals of 2–3 years for patients with Spigelman stage II and 1–2 years for patients with Spigelman stage III [46]. Chemopreventive therapies should be well tolerated and have a low toxicity. During intervention period, up to 81% of patients reported at least one adverse event, and 24% of patients discontinued intervention due to adverse events. Altogether, it seems unrealistic to expect that the regimens under investigation in the present study, would be suitable as a life-time chemopreventive treatment.

In conclusion, high dose UDCA co-treatment completely counteracts the positive effect of celecoxib, namely the reduction of duodenal polyp density in patients with FAP. It still needs investigation whether low dose UDCA co-treatment does have a beneficial effects in this respect. The benefit of long term use of celecoxib for duodenal cancer prevention in patients with FAP needs to be weighed against the potential risk of (cardiovascular) adverse events. The search for effective chemopreventive strategies is ongoing and drugs of interest for patients with FAP include sulindac and difluoromethylornithine [48], curcumin and quercetin [49], and eicosapentaenoic acid [45]. Future research has to result in suitable chemopreventive treatment regimes to avoid radical duodenectomy or duodenal cancer.

## Abbreviations

AE: Adverse Event; AMC: Academic Medical Centre Amsterdam; APC: Adenomatous polyposis coli; β2M: β-2 microglobulin; CA: Cholic acid; CDCA: Chenodeoxycholic acid; COX: Cyclooxygenase; CTCAE: Common toxicity criteria for adverse events; EMC: Erasmus Medical Centre Rotterdam; FAP: Familial adenomatous polyposis; LUMC: Leiden University Medical Centre; NSAID: Non-steroidal anti-inflammatory drug; PSC: Primary sclerosing cholangitis; qPCR: Real-time quantitative polymerase chain reaction; RUNMC: Radboud university Nijmegen medical centre; UC: Ulcerative colitis; UDCA: Ursodeoxycholic acid; UMCG: University Medical Centre Groningen.

## Competing interests

The authors declare that they have no competing interests.

## Authors’ contributions

BWHvH: protocol writing, patient recruitment, conducting the study protocol, data collection, analysis, and interpreting, drafting the manuscript; HMJR: data collection and analysis, drafting the manuscript; MEV-B: data collection and analysis, drafting the manuscript; ED, EMHM-V, JD, JKK, AMJL: patient recruitment, conducting the study protocol, data collection, critical review of the manuscript; IDN: data collection, analysis, and interpreting, critical review of the manuscript; EK: study design and protocol writing, critical review of the manuscript; WHMP: study design and protocol writing, data interpreting, drafting the manuscript; FMN: study design and protocol writing, patient recruitment, conducting the study protocol, data collection and interpreting, drafting the manuscript. All authors read and approved the final manuscript.
